# Supplementary material for: DCATS: differential composition analysis for flexible single-cell experimental designs
Source: Genome Biol. 2023 Jun 26;24:151. doi: 10.1186/s13059-023-02980-3 (PMC10294334; doi:10.1186/s13059-023-02980-3)
Supplement: Supplementary file 1 — Additional file 1. Supplementary Table S1-S10 and Supplementary Figures S1-S14. Additional file 1 contains Supplementary Figures and Supplementary Tables. [file 13059_2023_2980_MOESM1_ESM.pdf]

1 Supplementary Figures and Tables

Different DCATS model

- 'wtoPhi_wtoEM' indicates using basic beta-binomial distribution without bias correction or fixing over-dispersion term.
- 'wtoPhi_emK' indicates without fixing the over dispersion term Phi but clustering bias corrected by KNN matrix.
- 'wtoPhi_emSVM' indicates without fixing the over dispersion term Phi but clustering bias corrected by SVM matrix.
- 'wtoPhi_emU' indicates without fixing the over dispersion term Phi but clustering bias corrected by uniform matrix.
- 'estPhi_wtoEM' indicates using fixing the over dispersion term Phi but no clustering bias correction.
- 'estPhi_emK' indicates using fixing the over dispersion term Phi and clustering bias corrected by KNN matrix.
- 'estPhi_emSVM' indicates using fixing the over dispersion term Phi and clustering bias corrected by SVM matrix.
- 'estPhi_emU' indicates using fixing the over dispersion term Phi and clustering bias corrected by uniform matrix.

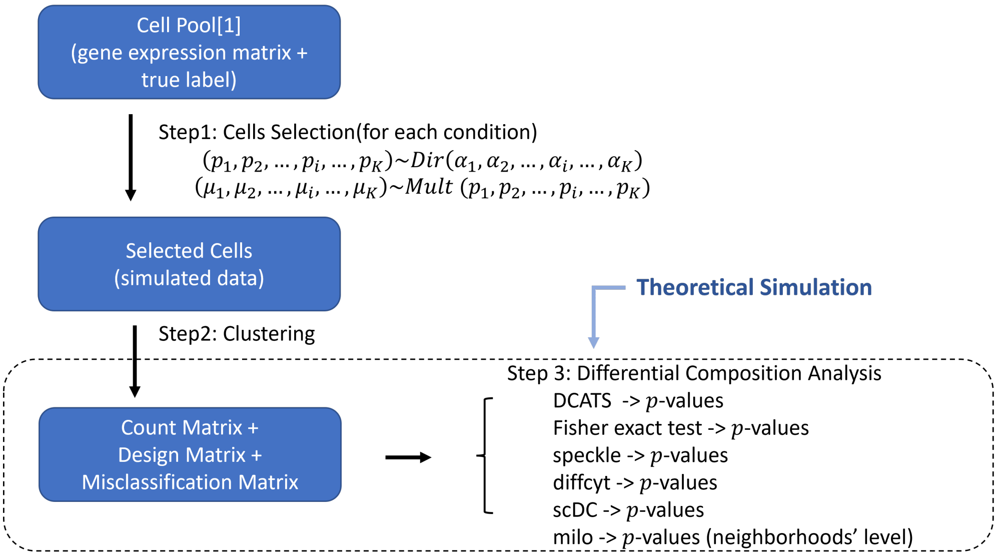

Fig. S1: Illustration of the simulation process.

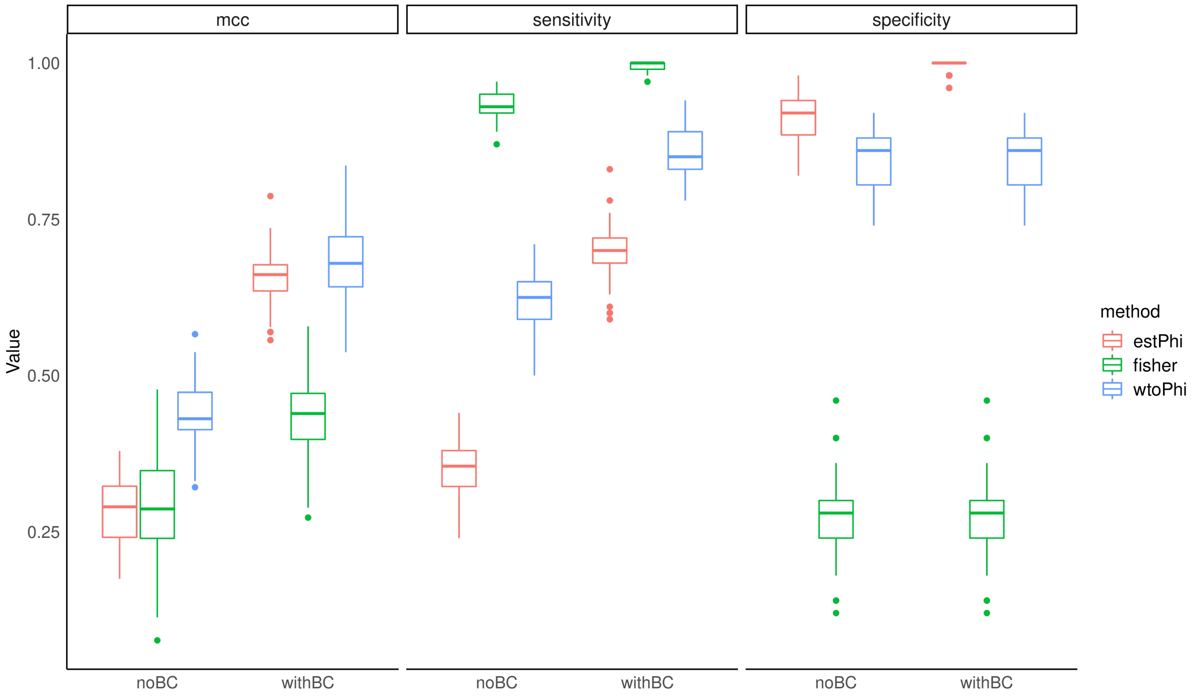

Fig. S2: The MCC, sensitivity, specificity of two DCATS models ('wtoPhi', 'estPhi') and fisher's exact test before and after bias correction.

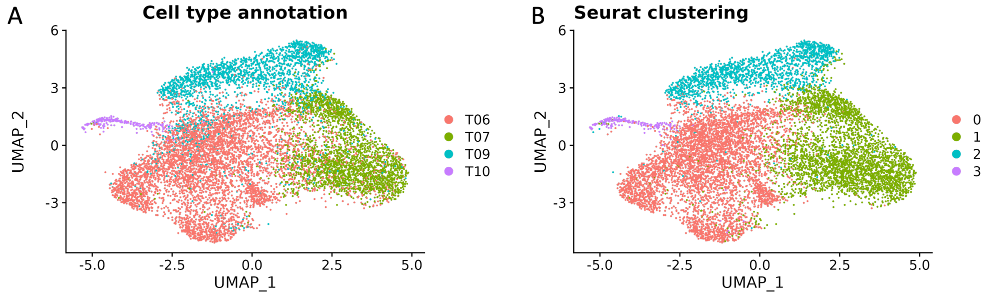

C

	T06/0	T07/1	T09/2	T10/3	RMSE
Origin	0.51760	0.24730	0.21240	0.02270	
noBC	0.47570	0.33780	0.16540	0.02110	0.1810
BC (empirical)	0.51756	0.24732	0.21242	0.02062	8.6722e-05
BC (knn)	0.53595	0.28012	0.16331	0.02063	0.1023
BC (svm)	0.55448	0.27955	0.15759	0.00837	0.1382

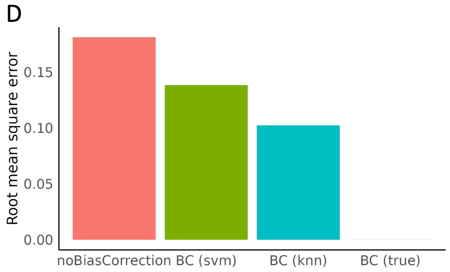

Fig. S3: DCATS enables bias correction. A) is the UMAP of the original identity defined by information coming from scRNA-seq and surface antibody staining. B) is the UMAP clustering given by Seurat. C) is the proportion of different cell types given by the paper, calculated by the seurat clustering results, or bias corrected by DCATS, and their root mean square error (RMSE) comparing to the origin proportion. D) Visualization of RMSE. 'BC (empirical)' indicates the bias-corrected proportion using the empirical confusion matrix. 'BC (knn)' indicates the bias-corrected proportion using the confusion matrix inferred by KNN graph. 'BC (svm)' indicates the bias-corrected proportion using the confusion matrix inferred by a support vector machine classifier.

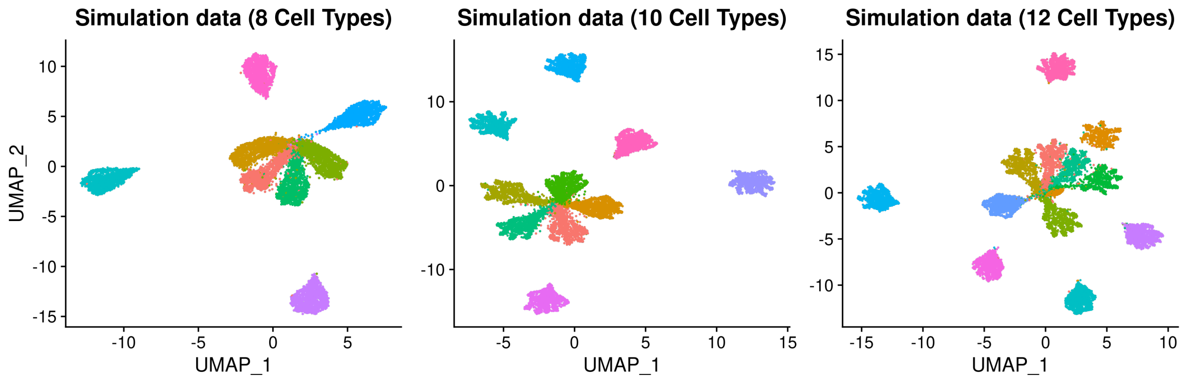

Fig. S4: The UMAP of cell pools with different numbers of cell types.

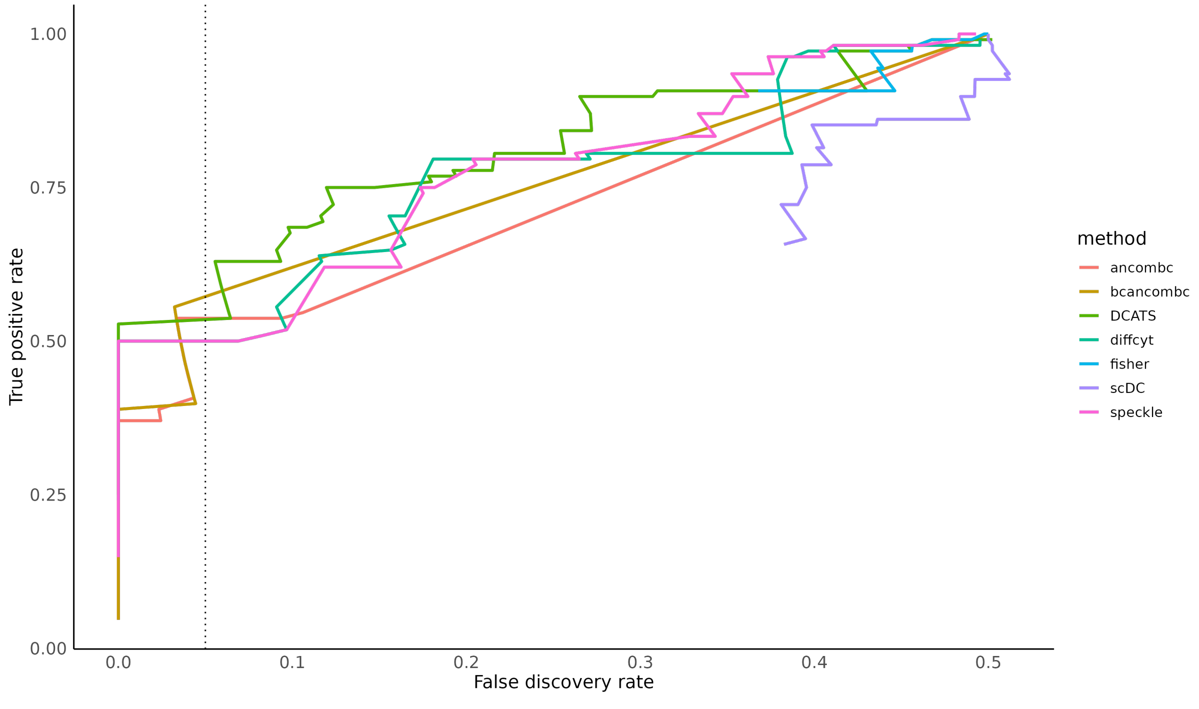

Fig. S5: The TPR vs FDR curve for simulation containing 8 cell types with 3 replicates in each condition. The dash black line indicates 0.05 FDR. N.B. As Milo provided the p-value in the 'neighbourhood' level, we didn't include the result of Milo here.

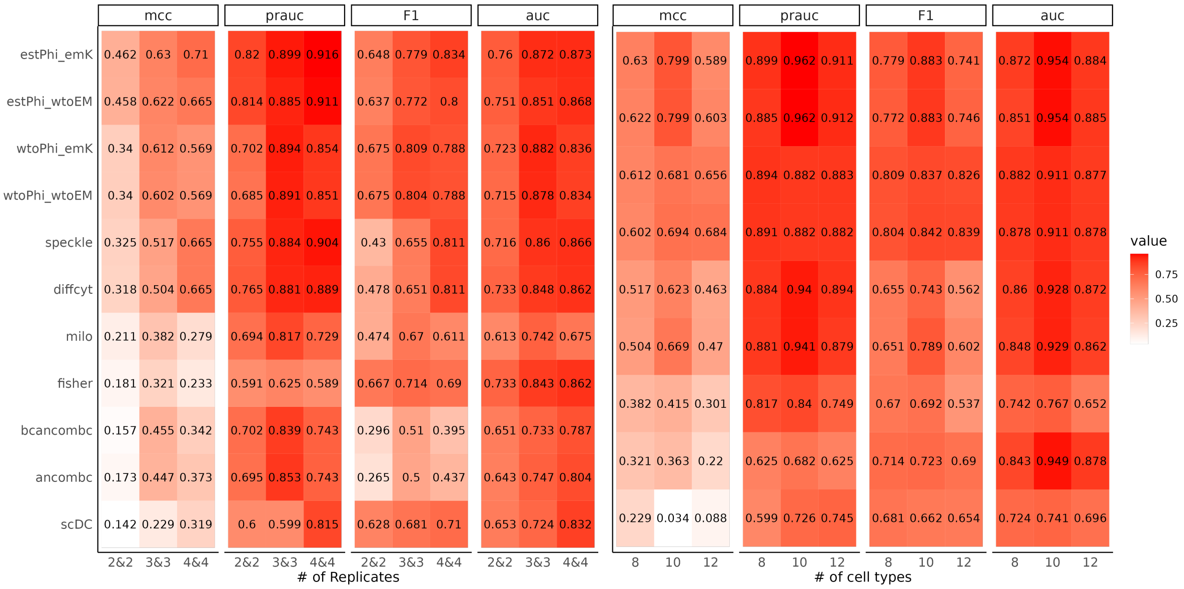

Fig. S6: The MCC, PRAUC, F1, AUC values of different DCATS models and other methods in different simulation settings. 'wtoPhi_emK' indicates using KNN matrix to do the bias correction without pre-estimated over-dispersion term. 'wtoPhi_wtoEM' indicates using basic beta-binomial distribution without bias correction and pre-estimated over-dispersion term. 'estPhi_emK' indicates using KNN matrix to do the bias correction with pre-estimated over-dispersion term. 'estPhi_wtoEM' indicates using pre-estimated over-dispersion term without bias correction.

	A	B	C	D	E	F	G	H
7	0.9925884	0.0000000	0.0074116	0.0000000	0.0000000	0.0000000	0.0000000	0.0000000
8	0.0000000	0.9996793	0.0003207	0.0000000	0.0000000	0.0000000	0.0000000	0.0000000
3	0.0000000	0.0000000	0.9616307	0.0004796	0.0163070	0.0100719	0.0000000	0.0115108
6	0.0016434	0.0000000	0.0061627	0.9921939	0.0000000	0.0000000	0.0000000	0.0000000
2	0.0000000	0.0000000	0.0557157	0.0019212	0.9178674	0.0033622	0.0000000	0.0211335
4	0.0000000	0.0000000	0.0507012	0.0016181	0.0253506	0.9099245	0.0000000	0.0124056
5	0.0000000	0.0000000	0.0012804	0.0000000	0.0000000	0.0000000	0.9987196	0.0000000
1	0.0000000	0.0000000	0.1407517	0.0007369	0.0324245	0.0140015	0.0000000	0.8120855

Fig. S7: The empirical confusion matrix calculated from ground truth and seurat clustering result in one simulation with default setting.

	A	B	C	D	E	F	G	H
A	0.9985960	0.0000263	0.0010624	0.0000622	0.0001555	0.0000641	0.0000173	0.0000162
B	0.0000287	0.9991608	0.0006151	0.0000152	0.0001598	0.0000000	0.0000203	0.0000000
C	0.0018297	0.0009675	0.9375284	0.0021082	0.0187538	0.0094317	0.0006555	0.0287252
D	0.0000951	0.0000213	0.0018715	0.9972366	0.0001855	0.0003109	0.0000653	0.0002138
E	0.0003277	0.0003075	0.0229506	0.0002558	0.9404613	0.0119533	0.0000271	0.0237168
F	0.0001565	0.0000000	0.0133674	0.0004963	0.0138433	0.9546358	0.0000113	0.0174892
G	0.0000433	0.0000464	0.0009505	0.0001067	0.0000321	0.0000116	0.9987777	0.0000316
H	0.0000565	0.0000000	0.0582963	0.0004888	0.0393305	0.0250432	0.0000443	0.8767404

Fig. S8: The estimated knn similarity matrix calculated from ground truth and seurat clustering result in one simulation with default setting.

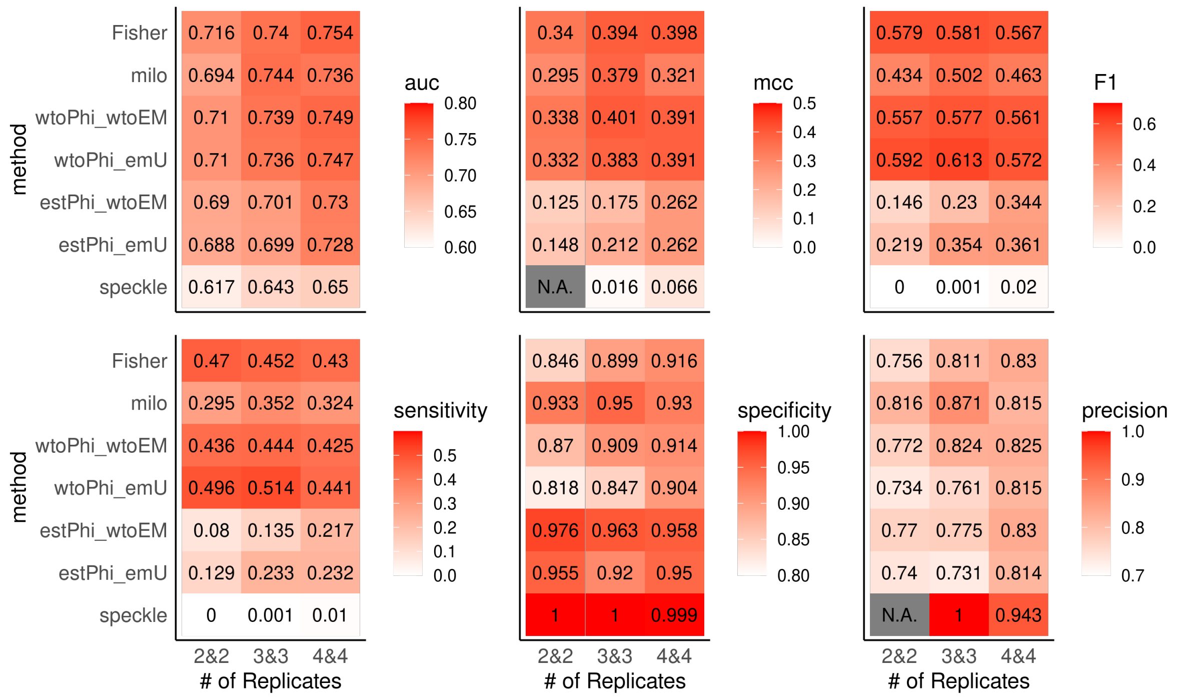

Fig. S9: The AUC, MCC, F1, sensitivity, specificity and precision values of different DCATS models and other methods in simulation with different numbers of biological replicates. 'wtoPhi_emU' indicates using uniform matrix to do the bias correction without pre-estimated over-dispersion term. 'wtoPhi_wtoEM' indicates using basic beta-binomial distribution without bias correction and pre-estimated over-dispersion term. 'estPhi_emU' indicates using uniform matrix to do the bias correction with pre-estimated over-dispersion term. 'estPhi_wtoEM' indicates using pre-estimated over-dispersion term without bias correction.

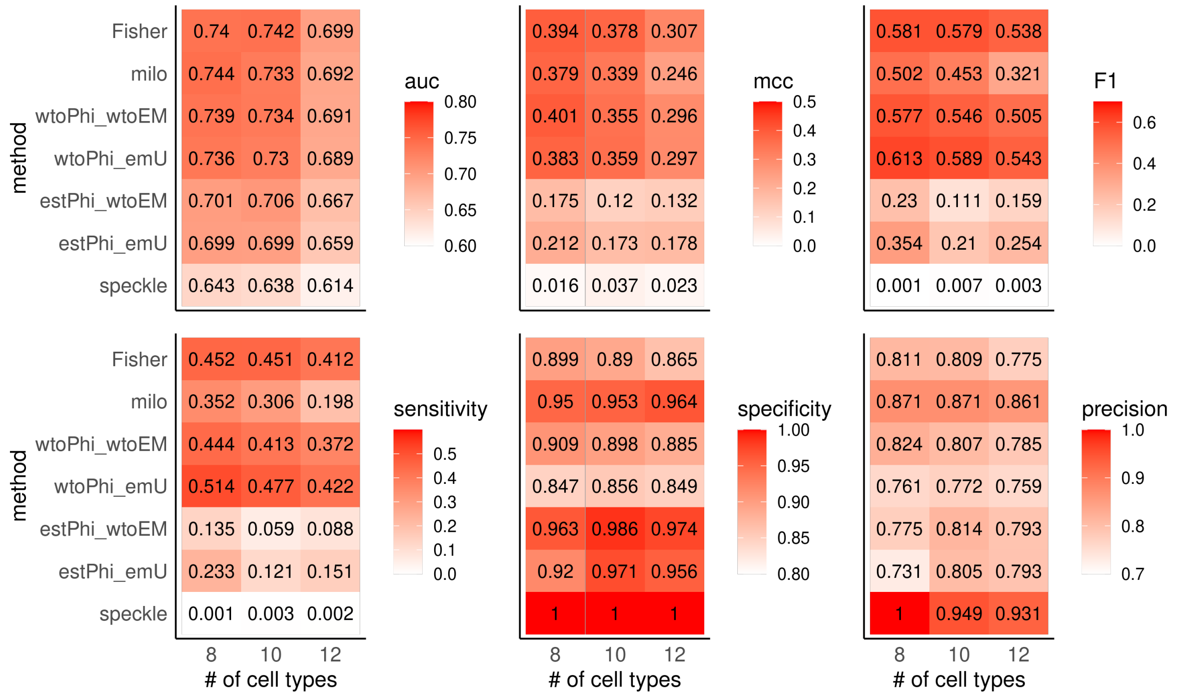

Fig. S10: The AUC, MCC, F1, sensitivity, specificity and precision values of different DCATS models and other methods in simulation with different numbers of cell types. As speckle didn't detect any neighborhood which has significant proportion difference with 12 cell types scenario, the MCC and precision is 'N.A.'.

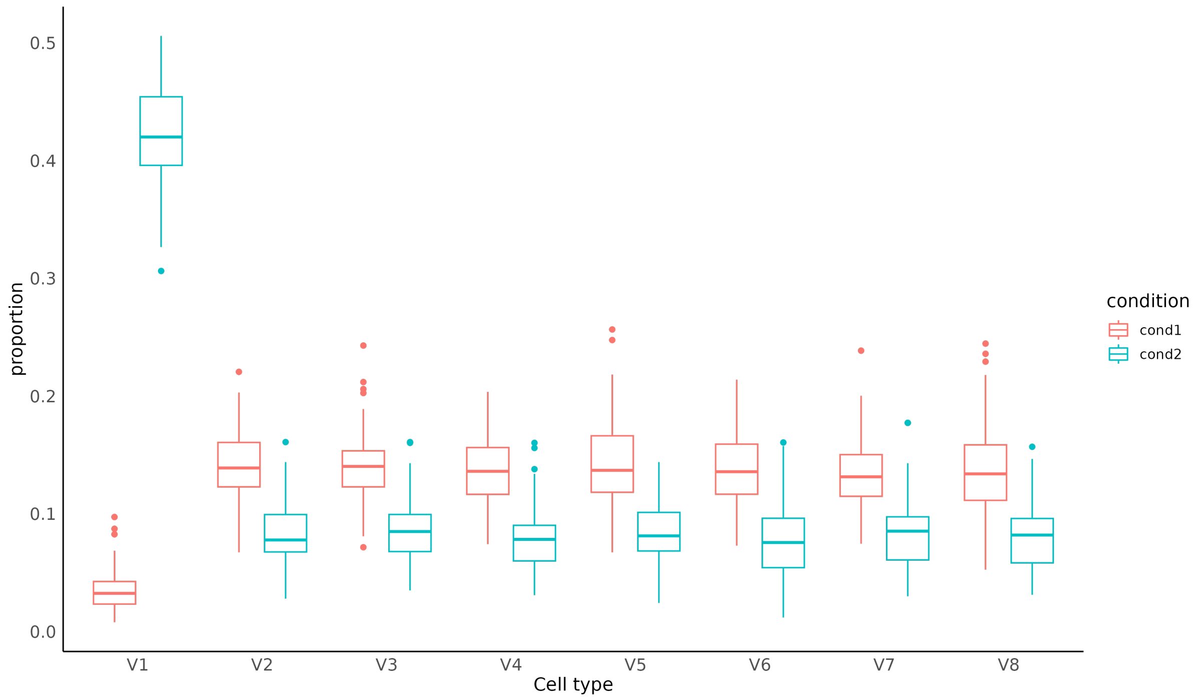

Fig. S11: The box plots of each cell type's simulated cell numbers in the simulation starting from counts.

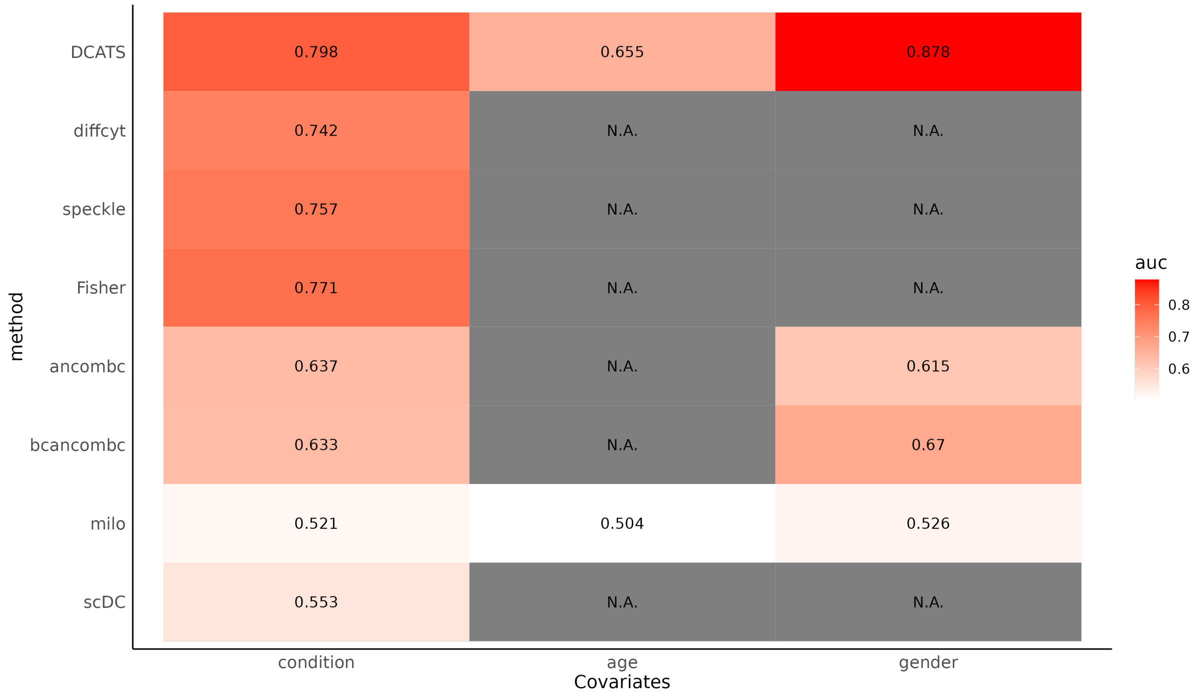

Fig. S12: The AUC values of different DCATS models and other methods in the simulation with confounding covariates. 'N.A.' means not applicable (same in the following plot).

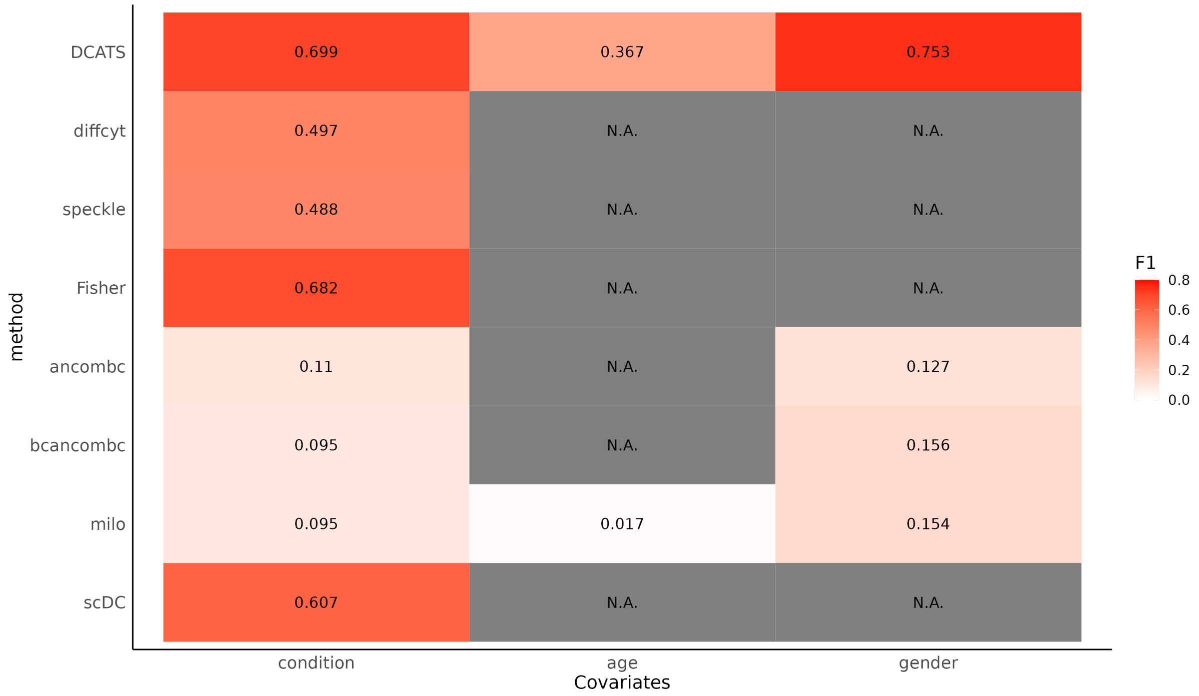

Fig. S13: The F1 values of different DCATS models and other methods in the simulation with confounding covariates.

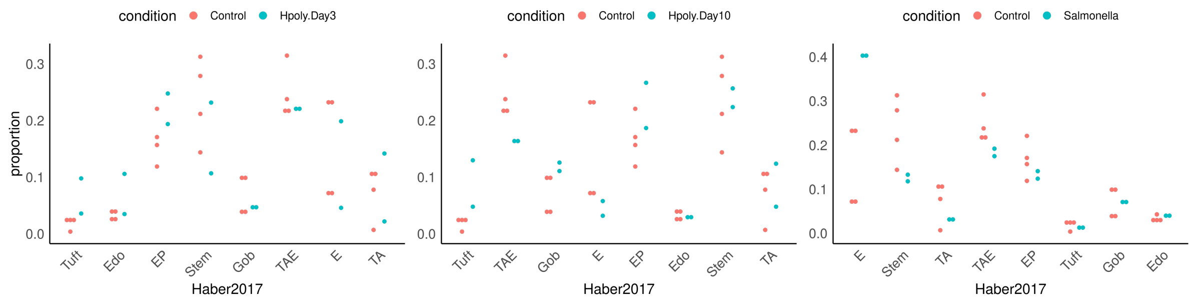

Fig. S14: The corrected cell type proportions of the Haber dataset obtained from using DCATS for bias correction

method	MCC	AUC	PRAUC	sensitivity	specificity	F1	replicates
estPhi_emK	**0.462**	**0.76**	**0.82**	0.527	0.902	0.648	2&2
estPhi_wtoEM	0.458	0.751	0.814	0.509	0.911	0.637	2&2
wtoPhi_emK	0.34	0.723	0.702	0.688	0.652	**0.675**	2&2
wtoPhi_wtoEM	0.34	0.715	0.685	0.688	0.652	**0.675**	2&2
speckle	0.325	0.716	0.755	0.286	**0.955**	0.43	2&2
diffcyt	0.318	0.733	0.765	0.339	0.92	0.478	2&2
milo	0.211	0.613	0.694	0.366	0.821	0.474	2&2
fisher	0.181	0.733	0.591	**0.848**	0.304	0.667	2&2
ancombc	0.173	0.643	0.695	0.161	0.946	0.265	2&2
bcancombc	0.157	0.651	0.702	0.188	0.92	0.296	2&2
scDC	0.142	0.653	0.6	0.732	0.402	0.628	2&2
estPhi_emK	**0.63**	0.872	**0.899**	0.685	0.926	0.779	3&3
estPhi_wtoEM	0.622	0.851	0.885	0.676	0.926	0.772	3&3
wtoPhi_emK	0.612	**0.882**	0.894	0.824	0.787	**0.809**	3&3
wtoPhi_wtoEM	0.602	0.878	0.891	0.815	0.787	0.804	3&3
speckle	0.517	0.86	0.884	0.509	0.954	0.655	3&3
diffcyt	0.504	0.848	0.881	0.509	0.944	0.651	3&3
bcancombc	0.455	0.733	0.839	0.343	**1**	0.51	3&3
ancombc	0.447	0.747	0.853	0.333	**1**	0.5	3&3
milo	0.382	0.742	0.817	0.63	0.75	0.67	3&3
fisher	0.321	0.843	0.625	**0.972**	0.25	0.714	3&3
scDC	0.229	0.724	0.599	0.861	0.333	0.681	3&3
estPhi_emK	**0.71**	**0.873**	**0.916**	0.759	0.94	**0.834**	4&4
estPhi_wtoEM	0.665	0.868	0.911	0.707	0.94	0.8	4&4
speckle	0.665	0.866	0.904	0.741	0.914	0.811	4&4
diffcyt	0.665	0.862	0.889	0.741	0.914	0.811	4&4
wtoPhi_emK	0.569	0.836	0.854	0.802	0.767	0.788	4&4
wtoPhi_wtoEM	0.569	0.834	0.851	0.802	0.767	0.788	4&4
ancombc	0.373	0.804	0.743	0.284	**0.983**	0.437	4&4
bcancombc	0.342	0.787	0.743	0.25	**0.983**	0.395	4&4
scDC	0.319	0.832	0.815	0.888	0.388	0.71	4&4
milo	0.279	0.675	0.729	0.569	0.707	0.611	4&4
fisher	0.233	0.862	0.589	**0.922**	0.25	0.69	4&4

Table S1: MCC, AUC, PRAUC, sensitivity, specificity, and F1 for different tests and DCATS with the change of replicates numbers. 'replicates' indicates the number of biological replicates in each condition.

method	CI of MCC	CI of PRAUC	CI of F1	CI of AUC	replicates
DCATS	(0.352, 0.54)	(0.75, 0.854)	(0.564, 0.703)	(0.689, 0.812)	2&2
speckle	(0.212, 0.427)	(0.664, 0.802)	(0.322, 0.526)	(0.646, 0.767)	2&2
diffcyt	(0.206, 0.432)	(0.677, 0.809)	(0.368, 0.568)	(0.676, 0.789)	2&2
milo	(0.103, 0.332)	(0.605, 0.764)	(0.372, 0.571)	(0.559, 0.671)	2&2
Fisher	(0.053, 0.317)	(0.512, 0.642)	(0.589, 0.714)	(0.663, 0.795)	2&2
ancombc	(0.063, 0.291)	(0.555, 0.759)	(0.144, 0.36)	(0.573, 0.696)	2&2
bcancombc	(0.011, 0.286)	(0.563, 0.777)	(0.157, 0.403)	(0.57, 0.709)	2&2
scDC	(0.018, 0.266)	(0.528, 0.657)	(0.574, 0.68)	(0.59, 0.702)	2&2
DCATS	(0.515, 0.724)	(0.852, 0.934)	(0.702, 0.835)	(0.83, 0.913)	3&3
speckle	(0.418, 0.592)	(0.84, 0.922)	(0.577, 0.725)	(0.819, 0.903)	3&3
diffcyt	(0.398, 0.592)	(0.831, 0.92)	(0.576, 0.733)	(0.802, 0.896)	3&3
bcancombc	(0.379, 0.52)	(0.778, 0.891)	(0.431, 0.606)	(0.504, 0.813)	3&3
ancombc	(0.382, 0.527)	(0.792, 0.883)	(0.411, 0.61)	(0.693, 0.82)	3&3
milo	(0.247, 0.519)	(0.766, 0.865)	(0.592, 0.74)	(0.678, 0.803)	3&3
Fisher	(0.227, 0.381)	(0.56, 0.678)	(0.651, 0.756)	(0.808, 0.898)	3&3
scDC	(0.107, 0.363)	(0.517, 0.671)	(0.602, 0.729)	(0.659, 0.791)	3&3
DCATS	(0.625, 0.801)	(0.883, 0.938)	(0.775, 0.883)	(0.834, 0.908)	4&4
diffcyt	(0.566, 0.748)	(0.834, 0.934)	(0.745, 0.854)	(0.824, 0.904)	4&4
speckle	(0.553, 0.749)	(0.864, 0.938)	(0.739, 0.858)	(0.832, 0.902)	4&4
ancombc	(0.315, 0.451)	(0.672, 0.895)	(0.365, 0.52)	(0.762, 0.851)	4&4
bcancombc	(0.27, 0.41)	(0.633, 0.888)	(0.306, 0.468)	(0.741, 0.834)	4&4
scDC	(0.189, 0.428)	(0.744, 0.854)	(0.638, 0.762)	(0.78, 0.879)	4&4
milo	(0.144, 0.399)	(0.608, 0.793)	(0.529, 0.681)	(0.594, 0.741)	4&4
Fisher	(0.147, 0.349)	(0.516, 0.641)	(0.64, 0.734)	(0.818, 0.893)	4&4

Table S2: The confidence interval of MCC, AUC, F1, and PRAUC for different tests and DCATS with the change of replicates numbers.. 'replicates' indicates the number of biological replicates in each condition.

method	MCC	AUC	PRAUC	sensitivity	specificity	F1	clustersN
estPhi_emK	**0.63**	0.872	**0.899**	0.685	0.926	0.779	8
estPhi_wtoEM	0.622	0.851	0.885	0.676	0.926	0.772	8
wtoPhi_emK	0.612	**0.882**	0.894	0.824	0.787	**0.809**	8
wtoPhi_wtoEM	0.602	0.878	0.891	0.815	0.787	0.804	8
speckle	0.517	0.86	0.884	0.509	0.954	0.655	8
diffcyt	0.504	0.848	0.881	0.509	0.944	0.651	8
bcancombc	0.455	0.733	0.839	0.343	**1**	0.51	8
ancombc	0.447	0.747	0.853	0.333	**1**	0.5	8
milo	0.382	0.742	0.817	0.63	0.75	0.67	8
fisher	0.321	0.843	0.625	**0.972**	0.25	0.714	8
scDC	0.229	0.724	0.599	0.861	0.333	0.681	8
estPhi_wtoEM	**0.799**	**0.954**	**0.962**	0.807	0.98	**0.883**	10
estPhi_emK	0.799	0.954	0.962	0.807	0.98	**0.883**	10
wtoPhi_wtoEM	0.694	0.911	0.882	0.82	0.873	0.842	10
wtoPhi_emK	0.681	0.911	0.882	0.82	0.86	0.837	10
diffcyt	0.669	0.929	0.941	0.673	0.967	0.789	10
speckle	0.623	0.928	0.94	0.607	0.973	0.743	10
milo	0.415	0.767	0.84	0.66	0.753	0.692	10
ancombc	0.378	0.783	0.857	0.267	**0.993**	0.419	10
bcancombc	0.373	0.8	0.857	0.26	**0.993**	0.411	10
fisher	0.363	0.949	0.682	**1**	0.233	0.723	10
scDC	0.034	0.741	0.726	0.967	0.047	0.662	10
wtoPhi_wtoEM	**0.684**	0.878	0.882	0.822	0.861	**0.839**	12
wtoPhi_emK	0.656	0.877	0.883	0.817	0.839	0.826	12
estPhi_wtoEM	0.603	**0.885**	**0.912**	0.628	0.944	0.746	12
estPhi_emK	0.589	0.884	0.911	0.628	0.933	0.741	12
diffcyt	0.47	0.862	0.879	0.45	0.956	0.602	12
speckle	0.463	0.872	0.894	0.4	0.978	0.562	12
bcancombc	0.325	0.732	0.842	0.206	**0.994**	0.339	12
milo	0.301	0.652	0.749	0.422	0.85	0.537	12
ancombc	0.293	0.736	0.754	0.172	**0.994**	0.292	12
fisher	0.22	0.878	0.625	**0.95**	0.194	0.69	12
scDC	0.088	0.696	0.745	0.889	0.172	0.654	12

Table S3: MCC, AUC, sensitivity, specificity and F1 for different tests and DCATS with different numbers of cell types in samples (keep only three significant decimal digits). 'clustersN' indicates the number of cell types in samples. N.B. the actual values about 'estPhi_emK' and 'estPhi_wtoEM' are close to each other but not the same.

method	CI of MCC	CI of PRAUC	CI of F1	CI of AUC	replicates
DCATS	(0.515, 0.724)	(0.852, 0.934)	(0.702, 0.835)	(0.83, 0.913)	8
speckle	(0.418, 0.592)	(0.84, 0.922)	(0.577, 0.725)	(0.819, 0.903)	8
diffcyt	(0.398, 0.592)	(0.831, 0.92)	(0.576, 0.733)	(0.802, 0.896)	8
bcancombc	(0.379, 0.52)	(0.778, 0.891)	(0.431, 0.606)	(0.504, 0.813)	8
ancombc	(0.382, 0.527)	(0.792, 0.883)	(0.411, 0.61)	(0.693, 0.82)	8
milo	(0.247, 0.519)	(0.766, 0.865)	(0.592, 0.74)	(0.678, 0.803)	8
Fisher	(0.227, 0.381)	(0.56, 0.678)	(0.651, 0.756)	(0.808, 0.898)	8
scDC	(0.107, 0.363)	(0.517, 0.671)	(0.602, 0.729)	(0.659, 0.791)	8
DCATS	(0.738, 0.855)	(0.943, 0.972)	(0.845, 0.917)	(0.934, 0.965)	10
diffcyt	(0.612, 0.734)	(0.918, 0.956)	(0.744, 0.829)	(0.908, 0.946)	10
speckle	(0.559, 0.684)	(0.915, 0.957)	(0.69, 0.786)	(0.903, 0.954)	10
milo	(0.342, 0.527)	(0.801, 0.874)	(0.64, 0.747)	(0.73, 0.809)	10
ancombc	(0.324, 0.444)	(0.812, 0.886)	(0.354, 0.496)	(0.752, 0.824)	10
bcancombc	(0.322, 0.436)	(0.815, 0.887)	(0.34, 0.486)	(0.762, 0.834)	10
Fisher	(0.323, 0.428)	(0.622, 0.746)	(0.684, 0.77)	(0.928, 0.963)	10
scDC	(-0.058, 0.132)	(0.683, 0.794)	(0.611, 0.709)	(0.696, 0.787)	10
DCATS	(0.506, 0.664)	(0.88, 0.935)	(0.67, 0.787)	(0.85, 0.925)	12
diffcyt	(0.411, 0.519)	(0.828, 0.905)	(0.541, 0.659)	(0.813, 0.901)	12
speckle	(0.401, 0.51)	(0.859, 0.921)	(0.492, 0.623)	(0.831, 0.914)	12
bcancombc	(0.263, 0.367)	(0.724, 0.87)	(0.245, 0.398)	(0.671, 0.776)	12
milo	(0.201, 0.372)	(0.695, 0.794)	(0.476, 0.589)	(0.606, 0.687)	12
ancombc	(0.225, 0.334)	(0.719, 0.861)	(0.198, 0.357)	(0.654, 0.775)	12
Fisher	(0.151, 0.314)	(0.58, 0.687)	(0.653, 0.736)	(0.842, 0.918)	12
scDC	(0.018, 0.158)	(0.707, 0.783)	(0.607, 0.691)	(0.656, 0.741)	12

Table S4: The confidence interval of MCC, AUC, F1, and PRAUC for different tests and DCATS with different numbers of cell types in samples (keep only three significant decimal digits). 'clustersN' indicates the number of cell types in samples.

	Cell type1	Cell type2	Cell type3	Cell type4	Cell type5	Cell type6	Cell type7	Cell type8
Condition1	8	8	8	8	8	8	8	8
Condition2	6	6	10	10	8	8	8	8
Age(per year)	-0.05	+0.05	0	0	-0.05	+0.05	0	0
Gender	-2	+2	0	0	-2	+2	0	0

Table S5: The count units of different conditions and the confounding effects of age and gender.

methods	MCC	AUC	PRAUC	sensitivity	specificity	precision	F1
ancombc	0.965	0.999	1	1	0.991	0.939	0.969
bcancombc	0.965	0.999	1	1	0.991	0.939	0.969
dcats_defRef	0.948	1	0.969	1	0.986	0.912	0.954
dcats_autoRef	0.889	1	1	1	0.968	0.816	0.899
DCATS	0.762	0.999	1	1	0.917	0.633	0.775
speckle	0.273	0.999	0.705	1	0.392	0.19	0.32
diffcyt	0.273	0.998	0.564	1	0.392	0.19	0.32
wtoPhi_emK	0.19	0.996	0.534	1	0.23	0.157	0.271
milo	0.151	0.999	N.A.	1	0.157	0.145	0.253
Fisher	0.034	1	1	1	0.009	0.126	0.224
scDC	N.A.	0.041	0.126	1	0	0.125	0.222

Table S6: The MCC, AUC, PRAUC, sensitivity, precision, specificity, and F1 for simulation starting from count.

cluster	truth	wtoPhi_wtoEM	wtoPhi_emSVM	estPhi_wtoEM	estPhi_emSVM	fisher	scDC	speckle	milo_pct
B cells	N	0.997	0.997	0.995	0.995	0.338	0	0.989	0.032
CD14+ Monocytes	N	0.51	0.51	0.537	0.527	0.001	0.48	0.989	0.186
CD4 T cells	N	0.953	0.953	0.926	0.921	0.252	0.408	0.989	0.058
CD8 T cells	N	0.734	0.734	0.581	0.566	0.008	0.348	0.989	0.187
Dendritic cells	N	0.496	0.496	0.815	0.96	0.252	0.333	0.989	0
FCGR3A+ Monocytes	N	0.603	0.603	0.729	0.335	0.008	0.077	0.989	0.101
Megakaryocytes	N	0.738	0.738	0.82	0.364	0.862	0.761	0.989	0.167
NK cells	N	0.244	0.244	0.514	0.508	0	0.018	0.989	0.071

Table S7: The percentages of differential abundance neighborhoods given by milo and p-values given by other methods for Kang dataset.

Cell type	DCATS (no reference)	DCATS (club reference)
**Alveolar macrophage**	**0.0021**	0.0849
B cells	0.0038	0.0068
Capillary endothelial cells	0.033	0.0141
Ccl17+/Cd103-/Cd11b- dendritic cells	0.6332	0.1099
Cd103+/Cd11b- dendritic cells	0.5535	0.1916
CD209+/Cd11b+ dendritic cells	0.4379	0.1819
**Cd4+ T cells**	**0.0109**	0.005
CD8+ T cells	0.0671	0.0079
**Ciliated cells**	0.8631	**0.0053**
classical monocyte (Ly6c2+)	0.0001	0.0001
Club cells	0.066	1
Eosinophils	0.0207	0.0121
Fn1+ macrophage	0.3597	0.0341
Gamma-Delta T cells	1	0.1324
Goblet cells	0.5168	0.0091
Interstitial Fibroblast	0.0697	0.9195
Interstitial macrophages	0.082	0.0035
Lipofibroblast	0.3004	0.6183
low quality cells	0.2152	0.0186
lymphatic endothelial cells	0.2415	0.3332
Megakaryocytes	0.0019	0.002
Mesothelial cells	0.6881	0.0036
Mki67+ proliferating cells	0.0556	0.4807
Natural Killer cells	0.6935	0.0209
Neutrophils	0.6669	0.0614
non-classical monocyte (Ly6c2-)	0.0051	0.0061
Plasma cells	0.009	0.0136
red blood cells	0.2999	0.1496
Smooth muscle cells	0.1801	0.4979
**Type2 pneumocytes**	**0.0496**	0.2228
Type1 pneumocytes	0.6713	0.4557
vascular endothelial cells	0.0578	0.0222
Vcam1+ endothelial cells	0.154	0.5367

Table S8: The p-value calculated by DCATS without any reference group and DCATS using club cells as the reference group.

	Edo	E	EP	Gob	Stem	TA	TAE	Tuft
Edo	0.9785933	0.0006868	0.0005495	0.0093209	0.0102934	0.0114537	0.0087549	0
E	0	0.9800824	0.017033	0	0	0	0	0
EP	0	0.018544	0.9401099	0	0.0005147	0.0572687	0.0087549	0
Gob	0.0091743	0	0	0.9826897	0.0056613	0.0017621	0.0019455	0
Stem	0.0030581	0.0006868	0	0.0026631	0.8625836	0.0933921	0.0345331	0
TA	0	0	0.0335165	0	0.0761709	0.7506608	0.0617704	0
TAE	0	0	0.0082418	0.0039947	0.0303654	0.0748899	0.8793774	0
Tuft	0.0091743	0	0.0005495	0.0013316	0.0144107	0.0105727	0.0048638	1

Table S9: The confusion matrix used for the Haber dataset.

cluster	truth	wtoPhi_wtoEM	wtoPhi_emSVM	estPhi_wtoEM	estPhi_emSVM	fisher	scDC	speckle	milo_pct	treatment
Endocrine	N	0.13	0.13	0.288	0.273	0	0.36	0.714	0	Hpoly.Day3
Enterocyte	N	0.641	0.641	0.448	0.41	0	0.002	0.748	0.071	Hpoly.Day3
Enterocyte.Progenitor	N	0.103	0.103	0.315	0.278	0	0.341	0.714	0.067	Hpoly.Day3
Goblet	N	0.437	0.437	0.589	0.571	0.002	0.003	0.748	0	Hpoly.Day3
Stem	N	0.254	0.254	0.222	0.164	0.032	0.01	0.714	0	Hpoly.Day3
TA	N	0.883	0.883	0.883	0.975	0.205	0.099	0.993	0	Hpoly.Day3
TA.Early	N	0.288	0.288	0.64	0.612	0.002	0.006	0.781	0.088	Hpoly.Day3
Tuft	P	0.04	0.04	0.07	0.061	0	0.069	0.714	0	Hpoly.Day3
Endocrine	N	0.39	0.39	0.925	0.867	0.379	0.035	0.923	0	Hpoly.Day10
Enterocyte	P	0.091	0.091	0.009	0.006	0	0.002	0.149	0.472	Hpoly.Day10
Enterocyte.Progenitor	N	0.132	0.132	0.285	0.233	0	0.103	0.349	0.162	Hpoly.Day10
Goblet	P	0.051	0.051	0.116	0.105	0	0.009	0.235	0.217	Hpoly.Day10
Stem	N	0.824	0.824	0.865	0.83	0.279	0.406	0.923	0.096	Hpoly.Day10
TA	N	0.852	0.852	0.836	0.592	1	0.572	0.923	0.647	Hpoly.Day10
TA.Early	P	0.015	0.015	0.132	0.083	0	0.224	0.235	0.266	Hpoly.Day10
Tuft	P	0.02	0.02	0.013	0.01	0	0	0.112	0.5	Hpoly.Day10
Endocrine	N	0.426	0.426	0.798	0.784	0.429	0.84	0.761	0.308	Salmonella
Enterocyte	P	0.008	0.008	0	0	0	0.022	0.008	0.457	Salmonella
Enterocyte.Progenitor	N	0.146	0.146	0.561	0.495	0	0.357	0.647	0.019	Salmonella
Goblet	N	0.681	0.681	0.821	0.802	0.558	0.253	0.864	0.1	Salmonella
Stem	P	0.031	0.031	0.059	0.046	0	0.08	0.094	0.32	Salmonella
TA	P	0.026	0.026	0.192	0.318	0	0.801	0.216	0.538	Salmonella
TA.Early	P	0.03	0.03	0.261	0.268	0	0.39	0.216	0.015	Salmonella
Tuft	N	0.587	0.587	0.833	0.844	0.129	0.059	0.761	0	Salmonella

Table S10: The percentages of differential abundance neighborhoods given by milo and p-values given by other methods for Haber dataset.
